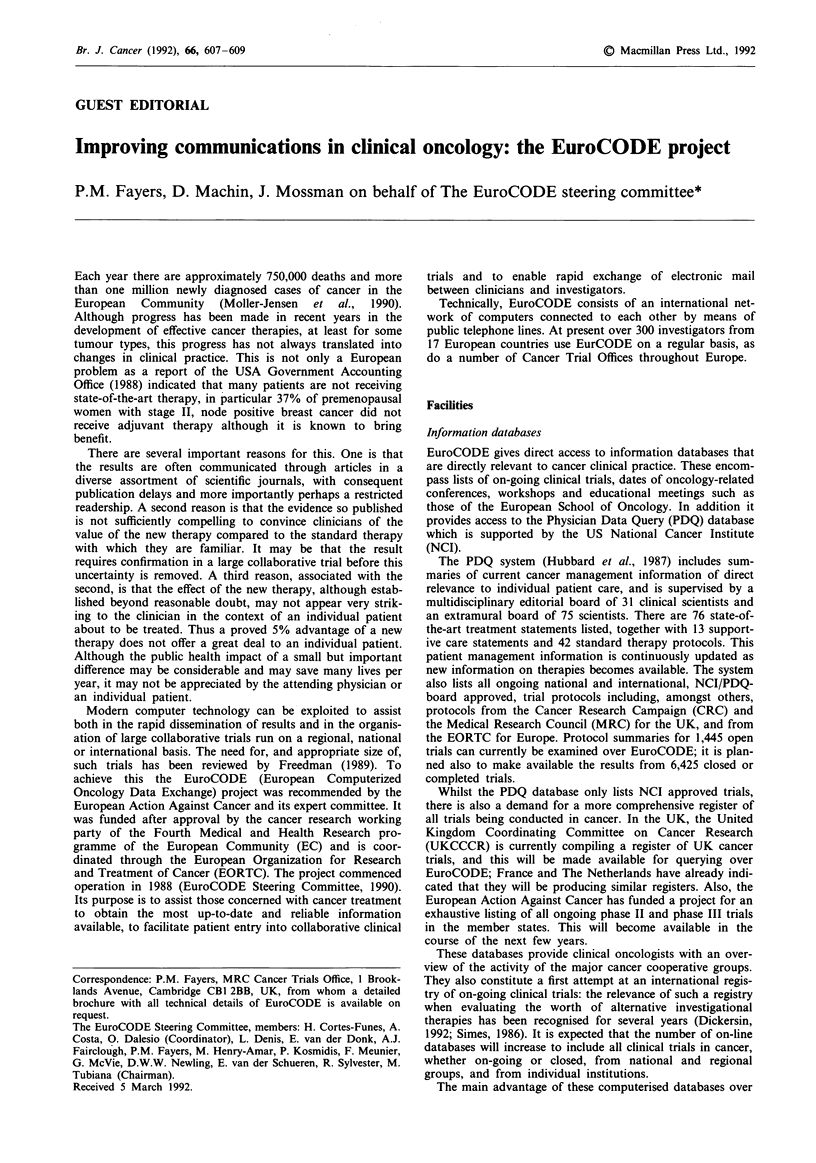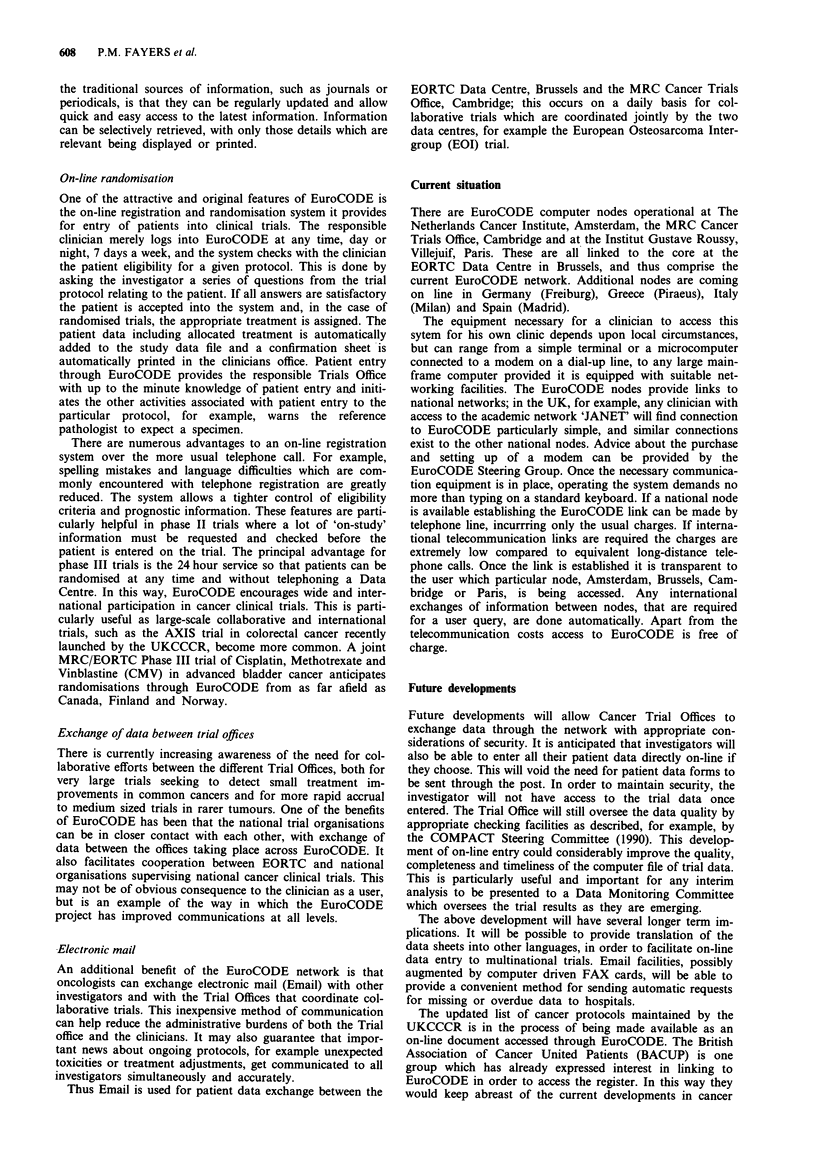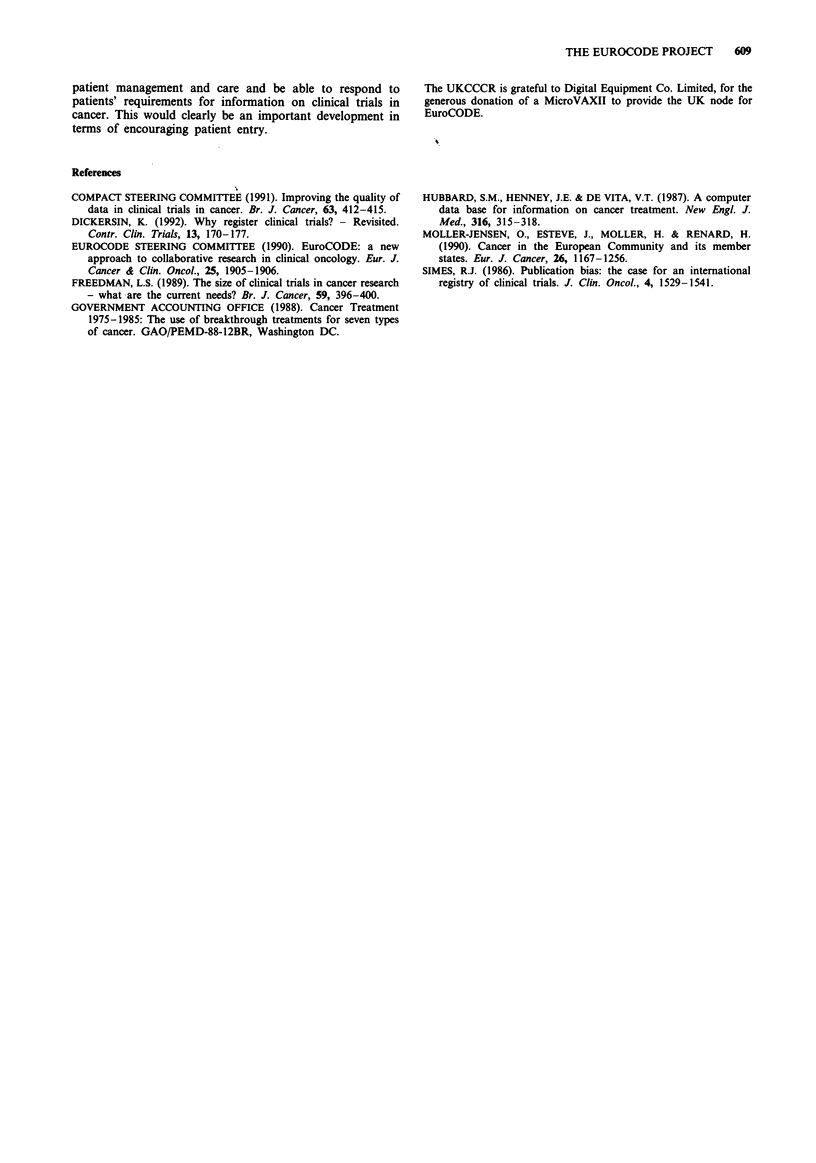# Improving communications in clinical oncology: the EuroCODE project. The EuroCODE Steering Committee.

**DOI:** 10.1038/bjc.1992.323

**Published:** 1992-10

**Authors:** P. M. Fayers, D. Machin, J. Mossman


Br. J. Cancer (1992), 66, 607-609 © Macmillan Press Ltd., 1992

GUEST EDITORIAL

### Improving communications in clinical oncology: the EuroCODE project

P.M. Fayers, D. Machin, J. Mossman on behalf of The EuroCODE steering committee*

Each year there are approximately 750,000 deaths and more than one million newly diagnosed cases of cancer in the European Community (Moller-Jensen *et al.*, 1990). Although progress has been made in recent years in the development of effective cancer therapies, at least for some tumour types, this progress has not always translated into changes in clinical practice. This is not only a European problem as a report of the USA Government Accounting Office (1988) indicated that many patients are not receiving state-of-the-art therapy, in particular 37% of premenopausal women with stage II, node positive breast cancer did not receive adjuvant therapy although it is known to bring benefit.

There are several important reasons for this. One is that the results are often communicated through articles in a diverse assortment of scientific journals, with consequent publication delays and more importantly perhaps a restricted readership. A second reason is that the evidence so published is not sufficiently compelling to convince clinicians of the value of the new therapy compared to the standard therapy with which they are familiar. It may be that the result requires confirmation in a large collaborative trial before this uncertainty is removed. A third reason, associated with the second, is that the effect of the new therapy, although established beyond reasonable doubt, may not appear very striking to the clinician in the context of an individual patient about to be treated. Thus a proved 5% advantage of a new therapy does not offer a great deal to an individual patient. Although the public health impact of a small but important difference may be considerable and may save many lives per year, it may not be appreciated by the attending physician or an individual patient.

Modern computer technology can be exploited to assist both in the rapid dissemination of results and in the organisation of large collaborative trials run on a regional, national or international basis. The need for, and appropriate size of, such trials has been reviewed by Freedman (1989). To achieve this the EuroCODE (European Computerized Oncology Data Exchange) project was recommended by the European Action Against Cancer and its expert committee. It was funded after approval by the cancer research working party of the Fourth Medical and Health Research programme of the European Community (EC) and is coordinated through the European Organization for Research and Treatment of Cancer (EORTC). The project commenced operation in 1988 (EuroCODE Steering Committee, 1990). Its purpose is to assist those concerned with cancer treatment to obtain the most up-to-date and reliable information available, to facilitate patient entry into collaborative clinical trials and to enable rapid exchange of electronic mail between clinicians and investigators.

Technically, EuroCODE consists of an international network of computers connected to each other by means of public telephone lines. At present over 300 investigators from 17 European countries use EurCODE on a regular basis, as do a number of Cancer Trial Offices throughout Europe.

### Facilities

### *Information databases*

EuroCODE gives direct access to information databases that are directly relevant to cancer clinical practice. These encompass lists of on-going clinical trials, dates of oncology-related conferences, workshops and educational meetings such as those of the European School of Oncology. In addition it provides access to the Physician Data Query (PDQ) database which is supported by the US National Cancer Institute (NCI).

The PDQ system (Hubbard *et al.*, 1987) includes summaries of current cancer management information of direct relevance to individual patient care, and is supervised by a multidisciplinary editorial board of 31 clinical scientists and an extramural board of 75 scientists. There are 76 state-of-the-art treatment statements listed, together with 13 supportive care statements and 42 standard therapy protocols. This patient management information is continuously updated as new information on therapies becomes available. The system also lists all ongoing national and international, NCI/PDQ-board approved, trial protocols including, amongst others, protocols from the Cancer Research Campaign (CRC) and the Medical Research Council (MRC) for the UK, and from the EORTC for Europe. Protocol summaries for 1,445 open trials can currently be examined over EuroCODE; it is planned also to make available the results from 6,425 closed or completed trials.

Whilst the PDQ database only lists NCI approved trials, there is also a demand for a more comprehensive register of all trials being conducted in cancer. In the UK, the United Kingdom Coordinating Committee on Cancer Research (UKCCCR) is currently compiling a register of UK cancer trials, and this will be made available for querying over EuroCODE; France and The Netherlands have already indicated that they will be producing similar registers. Also, the European Action Against Cancer has funded a project for an exhaustive listing of all ongoing phase II and phase III trials in the member states. This will become available in the course of the next few years.

These databases provide clinical oncologists with an overview of the activity of the major cancer cooperative groups. They also constitute a first attempt at an international registry of on-going clinical trials: the relevance of such a registry when evaluating the worth of alternative investigational therapies has been recognised for several years (Dickersin, 1992; Simes, 1986). It is expected that the number of on-line databases will increase to include all clinical trials in cancer, whether on-going or closed, from national and regional groups, and from individual institutions.

The main advantage of these computerised databases over

Correspondence: P.M. Fayers, MRC Cancer Trials Office, 1 Brooklands Avenue, Cambridge CB1 2BB, UK, from whom a detailed brochure with all technical details of EuroCODE is available on request.
The EuroCODE Steering Committee, members: H. Cortes-Funes, A. Costa, O. Dalesio (Coordinator), L. Denis, E. van der Donk, A.J. Fairclough, P.M. Fayers, M. Henry-Amar, P. Kosmidis, F. Meunier, G. McVie, D.W.W. Newling, E. van der Schueren, R. Sylvester, M. Tubiana (Chairman).
Received 5 March 1992.

the traditional sources of information, such as journals or periodicals, is that they can be regularly updated and allow quick and easy access to the latest information. Information can be selectively retrieved, with only those details which are relevant being displayed or printed.

*On-line randomisation*

One of the attractive and original features of EuroCODE is the on-line registration and randomisation system it provides for entry of patients into clinical trials. The responsible clinician merely logs into EuroCODE at any time, day or night, 7 days a week, and the system checks with the clinician the patient eligibility for a given protocol. This is done by asking the investigator a series of questions from the trial protocol relating to the patient. If all answers are satisfactory the patient is accepted into the system and, in the case of randomised trials, the appropriate treatment is assigned. The patient data including allocated treatment is automatically added to the study data file and a confirmation sheet is automatically printed in the clinicians office. Patient entry through EuroCODE provides the responsible Trials Office with up to the minute knowledge of patient entry and initiates the other activities associated with patient entry to the particular protocol, for example, warns the reference pathologist to expect a specimen.

There are numerous advantages to an on-line registration system over the more usual telephone call. For example, spelling mistakes and language difficulties which are commonly encountered with telephone registration are greatly reduced. The system allows a tighter control of eligibility criteria and prognostic information. These features are particularly helpful in phase II trials where a lot of 'on-study' information must be requested and checked before the patient is entered on the trial. The principal advantage for phase III trials is the 24 hour service so that patients can be randomised at any time and without telephoning a Data Centre. In this way, EuroCODE encourages wide and international participation in cancer clinical trials. This is particularly useful as large-scale collaborative and international trials, such as the AXIS trial in colorectal cancer recently launched by the UKCCCR, become more common. A joint MRC/EORTC Phase III trial of Cisplatin, Methotrexate and Vinblastine (CMV) in advanced bladder cancer anticipates randomisations through EuroCODE from as far afield as Canada, Finland and Norway.

*Exchange of data between trial offices*

There is currently increasing awareness of the need for collaborative efforts between the different Trial Offices, both for very large trials seeking to detect small treatment improvements in common cancers and for more rapid accrual to medium sized trials in rarer tumours. One of the benefits of EuroCODE has been that the national trial organisations can be in closer contact with each other, with exchange of data between the offices taking place across EuroCODE. It also facilitates cooperation between EORTC and national organisations supervising national cancer clinical trials. This may not be of obvious consequence to the clinician as a user, but is an example of the way in which the EuroCODE project has improved communications at all levels.

*Electronic mail*

An additional benefit of the EuroCODE network is that oncologists can exchange electronic mail (Email) with other investigators and with the Trial Offices that coordinate collaborative trials. This inexpensive method of communication can help reduce the administrative burdens of both the Trial office and the clinicians. It may also guarantee that important news about ongoing protocols, for example unexpected toxicities or treatment adjustments, get communicated to all investigators simultaneously and accurately.

Thus Email is used for patient data exchange between the EORTC Data Centre, Brussels and the MRC Cancer Trials Office, Cambridge; this occurs on a daily basis for collaborative trials which are coordinated jointly by the two data centres, for example the European Osteosarcoma Intergroup (EOI) trial.

**Current situation**

There are EuroCODE computer nodes operational at The Netherlands Cancer Institute, Amsterdam, the MRC Cancer Trials Office, Cambridge and at the Institut Gustave Roussy, Villejuif, Paris. These are all linked to the core at the EORTC Data Centre in Brussels, and thus comprise the current EuroCODE network. Additional nodes are coming on line in Germany (Freiburg), Greece (Piraeus), Italy (Milan) and Spain (Madrid).

The equipment necessary for a clinician to access this sytem for his own clinic depends upon local circumstances, but can range from a simple terminal or a microcomputer connected to a modem on a dial-up line, to any large mainframe computer provided it is equipped with suitable networking facilities. The EuroCODE nodes provide links to national networks; in the UK, for example, any clinician with access to the academic network 'JANET' will find connection to EuroCODE particularly simple, and similar connections exist to the other national nodes. Advice about the purchase and setting up of a modem can be provided by the EuroCODE Steering Group. Once the necessary communication equipment is in place, operating the system demands no more than typing on a standard keyboard. If a national node is available establishing the EuroCODE link can be made by telephone line, incurrring only the usual charges. If international telecommunication links are required the charges are extremely low compared to equivalent long-distance telephone calls. Once the link is established it is transparent to the user which particular node, Amsterdam, Brussels, Cambridge or Paris, is being accessed. Any international exchanges of information between nodes, that are required for a user query, are done automatically. Apart from the telecommunication costs access to EuroCODE is free of charge.

**Future developments**

Future developments will allow Cancer Trial Offices to exchange data through the network with appropriate considerations of security. It is anticipated that investigators will also be able to enter all their patient data directly on-line if they choose. This will void the need for patient data forms to be sent through the post. In order to maintain security, the investigator will not have access to the trial data once entered. The Trial Office will still oversee the data quality by appropriate checking facilities as described, for example, by the COMPACT Steering Committee (1990). This development of on-line entry could considerably improve the quality, completeness and timeliness of the computer file of trial data. This is particularly useful and important for any interim analysis to be presented to a Data Monitoring Committee which oversees the trial results as they are emerging.

The above development will have several longer term implications. It will be possible to provide translation of the data sheets into other languages, in order to facilitate on-line data entry to multinational trials. Email facilities, possibly augmented by computer driven FAX cards, will be able to provide a convenient method for sending automatic requests for missing or overdue data to hospitals.

The updated list of cancer protocols maintained by the UKCCCR is in the process of being made available as an on-line document accessed through EuroCODE. The British Association of Cancer United Patients (BACUP) is one group which has already expressed interest in linking to EuroCODE in order to access the register. In this way they would keep abreast of the current developments in cancer patient management and care and be able to respond to patients' requirements for information on clinical trials in cancer. This would clearly be an important development in terms of encouraging patient entry.

The UKCCCR is grateful to Digital Equipment Co. Limited, for the generous donation of a MicroVAXII to provide the UK node for EuroCODE.

**References**

COMPACT STEERING COMMITTEE (1991). Improving the quality of data in clinical trials in cancer. *Br. J. Cancer*, **63**, 412–415.

DICKERSIN, K. (1992). Why register clinical trials? – Revisited. *Contr. Clin. Trials*, **13**, 170–177.

EUROCODE STEERING COMMITTEE (1990). EuroCODE: a new approach to collaborative research in clinical oncology. *Eur. J. Cancer & Clin. Oncol.*, **25**, 1905–1906.

FREEDMAN, L.S. (1989). The size of clinical trials in cancer research – what are the current needs? *Br. J. Cancer*, **59**, 396–400.

GOVERNMENT ACCOUNTING OFFICE (1988). Cancer Treatment 1975–1985: The use of breakthrough treatments for seven types of cancer. GAO/PEMD-88-12BR, Washington DC.

HUBBARD, S.M., HENNEY, J.E. & DE VITA, V.T. (1987). A computer data base for information on cancer treatment. *New Engl. J. Med.*, **316**, 315–318.

MOLLER-JENSEN, O., ESTEVE, J., MOLLER, H. & RENARD, H. (1990). Cancer in the European Community and its member states. *Eur. J. Cancer*, **26**, 1167–1256.

SIMES, R.J. (1986). Publication bias: the case for an international registry of clinical trials. *J. Clin. Oncol.*, **4**, 1529–1541.